# Author Correction: Large spontaneous exchange bias in a weak ferromagnet Pb_6_Ni_9_(TeO_6_)_5_

**DOI:** 10.1038/s41598-019-42461-x

**Published:** 2019-05-03

**Authors:** B. Koteswararao, Tanmoy Chakrabarty, Tathamay Basu, Binoy Krishna Hazra, P. V. Srinivasarao, P. L. Paulose, S. Srinath

**Affiliations:** 1 Department of Physics, Indian Institute of Technology Tirupati, TIRUPATI, 517506 India; 2 School of Physics, University of Hyderabad, Hyderabad, 500046 India; 3 Tata Institute of Fundamental Research, Homi Bhabha Road, Colaba, Mumbai, 400005 India; 4 Laboratoire CRISMAT, UMR 6508 du CNRS et de l’Ensicaen, 6 Bd Marechal Juin, 14050 Caen, France; 5 S. S. & N. College, Narasaraopet, Guntur District, Andhra Pradesh, 522601 India

Correction to: *Scientific Reports* 10.1038/s41598-017-09056-w, published online 15 August 2017

In the original version of this Article, Binoy Krishna Hazra and S. Srinath were incorrectly affiliated with ‘Department of Physics, Indian Institute of Technology Tirupati, TIRUPATI, 517506, India’. The correct affiliation is listed below:

School of Physics, University of Hyderabad, Hyderabad, 500046, India

This error has now been corrected in the PDF and HTML versions of the Article, and in the accompanying Supplementary Information file.

