# Supplementary material for: Large spontaneous exchange bias in a weak ferromagnet Pb6Ni9(TeO6)5
Source: Sci Rep. 2017 Aug 15;7:8300. doi: 10.1038/s41598-017-09056-w (PMC5557810; doi:10.1038/s41598-017-09056-w)
Supplement: Supplementary file 1 — Supplementary Information: Large spontaneous exchange bias in a weak ferromagnet Pb6Ni9(TeO6)5 [file 41598_2017_9056_MOESM1_ESM.doc]

**Supplementary Information: *Large spontaneous exchange bias in a weak ferromagnet Pb_6_Ni_9_(TeO_6_)_5_***

B. Koteswararao,^1,2^ Tanmoy Chakrabarty,^3^ Tathamay Basu,^3,4^ Binoy Krishna Hazra,^2^ P. V. Srinivasarao,^5^ P. L. Paulose,^3^ S. Srinath^2^

*^1^Department of Physics, Indian Institute of Technology Tirupati, TIRUPATI, 517506, India.*

*^2^School of Physics, University of Hyderabad, Hyderabad, 500046, India.*

*^3^Tata Institute of Fundamental Research, Homi Bhabha Road, Colaba, Mumbai, 400005, India.*

*^4^Laboratoire CRISMAT, UMR 6508 du CNRS et de l’Ensicaen, 6 Bd Marechal*

*Juin, 14050, Caen, France.*

*^5^S. S. & N. College, Narasaraopet, Guntur District, Andhra Pradesh, 522601, India.*

Correspondence and requests for materials should be addressed to B.K. (email: [koteswararao@iittp.ac.in](mailto:koteswararao@iittp.ac.in) ) or S.S. (email: [srinath@uohyd.ac.in](mailto:srinath@uohyd.ac.in) )

**Rietveld refinement of powder X-ray diffraction**

**Figure S1**: Rietveld refinement on the powder XRD of Pb_6_Ni_9_(TeO_6_)_5_ samples.

**Table S I: The details of atomic positions of various atoms in Pb_6_Ni_9_(TeO_6_)_5_**

| Atom | Wyckoff position | *x/a* | *Y* | *Z* | Occupancy |
| --- | --- | --- | --- | --- | --- |
| Pb1 | *6g* | 0.73978 | 0.0 | 0.5 | 1.0 |
| Pb2 | *6g* | 0.0 | 0.38701 | 0.5 | 1.0 |
| Te1 | *4 f* | 0.6667 | 0.3333 | 0.60511 | 1.0 |
| Te2 | *6h* | 0.33775 | 0.16888 | 0.25 | 1.0 |
| Ni1 | *4f* | 0.6667 | 0.3333 | 0.39826 | 1.0 |
| Ni2 | *6h* | 0.16476 | -0.16476 | 0.25 | 1.0 |
| Ni3 | *6h* | 0.51023 | 0.48977 | 0.25 | 1.0 |
| Ni4 | *2b* | 0.0 | 0.0 | 0.25 | 1.0 |
| O1 | *12i* | 0.3357 | 0.0084 | 0.1716 | 1.0 |
| O2 | *12i* | 0.1779 | 0.0123 | 0.3281 | 1.0 |
| O3 | *12i* | 0.4843 | 0.3271 | 0.1697 | 1.0 |
| O4 | *12i* | 0.1704 | 0.3299 | 0.1644 | 1.0 |
| O5 | *12i* | 0.6537 | 0.1789 | 0.5025 | 1.0 |

**Table S II: The details of magnetic couplings in the honeycomb like layer.**

| Magnetic coupling path | Bond-length (Å) | Bond-angle (^o^) | Expected coupling type |
| --- | --- | --- | --- |
| Ni2-O2-Ni4 | 3.0 | 92.4 | FM |
| Ni2-O1-Ni3 | 2.91 | 90.7 | FM |
| Ni2-O4-Ni3 |  | 91.1 |  |
| Ni1-O3-Ni3 | 3.51 | 122.2 | AFM |


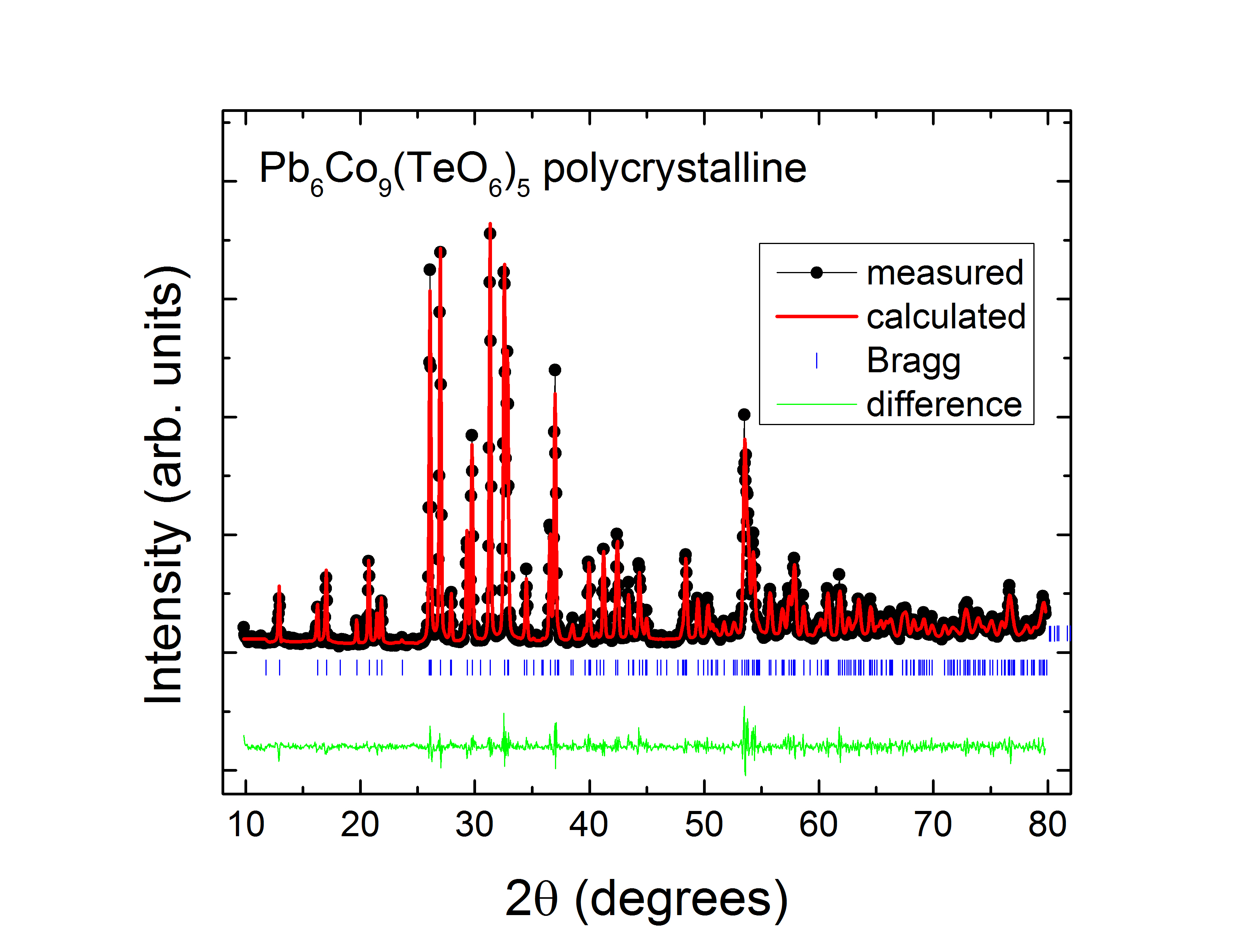


**Figure S2**: Rietveld refinement on the powder XRD of Pb_6_Co_9_(TeO_6_)_5_ samples.

**Table S III: The Comparison between the Reitveld refinement parameters of Pb_6_Ni_9_(TeO_6_)_5_ and Pb_6_Co_9_(TeO_6_)_5_ samples. It can be seen that the goodness of fit to the Co-sample is better than that of Ni-sample.**

| **Refinement Parameters** | Pb_6_Ni_9_(TeO_6_)_5_ | Pb_6_Co_9_(TeO_6_)_5_ |
| --- | --- | --- |
| R_p_ | 24.3 % | 16.7 % |
| R_wp_ | 22.3 % | 18.2 % |
| R_exp_ | 8.55 % | 13.5 % |
| χ^2^ | 6.83 | 1.81 |

**Table S IV: The parameters obtained from the magnetic data analysis of Pb_6_Ni_9_(TeO_6_)_5_ and Pb_6_Co_9_(TeO_6_)_5_ samples.**

| sample | C | μ_eff_ | θ_CW_ | *T*_N_ |
| --- | --- | --- | --- | --- |
| Pb_6_Ni_9_(TeO_6_)_5_ | 1.26 | 3.18 μ_B_ | -30 K | 25 K |
| Pb_6_Co_9_(TeO_6_)_5_ | 3.16 | 5.02 μ_B_ | -28 K | 26 K |
